# Sensitivity and Resilience to Predator Stress-Enhanced Ethanol Drinking Is Associated With Sex-Dependent Differences in Stress-Regulating Systems

**DOI:** 10.3389/fnbeh.2022.834880

**Published:** 2022-05-11

**Authors:** Mehrdad Alavi, Andrey E. Ryabinin, Melinda L. Helms, Michelle A. Nipper, Leslie L. Devaud, Deborah A. Finn

**Affiliations:** ^1^School of Pharmacy, Pacific University, Hillsboro, OR, United States; ^2^Department of Behavioral Neuroscience, Oregon Health and Science University, Portland, OR, United States; ^3^Department of Research, VA Portland Health Care System, Portland, OR, United States

**Keywords:** predator odor, corticosterone, C57BL/6J mice, hypothalamic-pituitary-adrenal axis, CRH, prefrontal cortex, hippocampus

## Abstract

Stress can increase ethanol drinking, and evidence confirms an association between post-traumatic stress disorder (PTSD) and the development of alcohol use disorder (AUD). Exposure to predator odor is considered a traumatic stressor, and predator stress (PS) has been used extensively as an animal model of PTSD. Our prior work determined that repeated exposure to intermittent PS significantly increased anxiety-related behavior, corticosterone levels, and neuronal activation in the hippocampus and prefrontal cortex in naïve male and female C57BL/6J mice. Intermittent PS exposure also increased subsequent ethanol drinking in a subgroup of animals, with heterogeneity of responses as seen with comorbid PTSD and AUD. The present studies built upon this prior work and began to characterize “sensitivity” and “resilience” to PS-enhanced drinking. Ethanol drinking was measured during baseline, intermittent PS exposure, and post-stress; mice were euthanized after 24-h abstinence. Calculation of median and interquartile ranges identified “sensitive” (>20% increase in drinking over baseline) and “resilient” (no change or decrease in drinking from baseline) subgroups. Intermittent PS significantly increased subsequent ethanol intake in 24% of male (↑60%) and in 20% of female (↑71%) C57BL/6J mice in the “sensitive” subgroup. Plasma corticosterone levels were increased significantly after PS in both sexes, but levels were lower in the “sensitive” vs. “resilient” subgroups. In representative mice from “sensitive” and “resilient” subgroups, prefrontal cortex and hippocampus were analyzed by Western Blotting for levels of corticotropin releasing factor (CRF) receptor 1, CRF receptor 2, CRF binding protein, and glucocorticoid receptor, vs. separate naïve age-matched mice. In prefrontal cortex, CRF receptor 1, CRF receptor 2, CRF binding protein, and glucocorticoid receptor levels were significantly higher in “sensitive” vs. naïve and “resilient” mice only in females. In hippocampus, CRF receptor 1, CRF receptor 2 and glucocorticoid receptor levels were significantly lower in “resilient” vs. naïve and “sensitive” mice across both sexes. These results indicate that sex strongly influences the effects of ethanol drinking and stress on proteins regulating stress and anxiety responses. They further suggest that targeting the CRF system and glucocorticoid receptors in AUD needs to consider the comorbidity of PTSD with AUD and sex of treated individuals.

## Introduction

Alcohol use disorder (AUD) encompasses both alcohol abuse and dependence, and it is considered a chronic brain disorder that is characterized by compulsive drinking, an inability to stop or to control alcohol use, and the presence of negative emotions when not drinking (NIAAA, 2021a,b). Some risk factors for developing AUD include binge drinking and heavy alcohol use over time, drinking at an early age, a range of mental health conditions that can be comorbid with AUD, and a history of trauma (NIAAA, 2021a). According to the 2019 National Survey on Drug Use and Health, 14.5 million people aged 12 and older (5.3% of this age group) had AUD, and deaths from alcohol-related causes made alcohol the third leading preventable cause of death in the United States (NIAAA, 2021b). Globally, alcohol misuse was the seventh leading risk factor for premature death and disability in 2016, accounting for 5.3% of all global deaths (∼3 million). However, among people aged 15–49, alcohol misuse was the first leading risk factor for premature death and disability (NIAAA, 2021b).

Post-traumatic stress disorder (PTSD) has been classified as a trauma- or stressor-related disorder (American Psychiatric Association, 2013). Individuals exposed to trauma display symptoms common to PTSD patients, but only a proportion of these individuals continue to exhibit post-trauma symptoms beyond 1 month and meet the diagnostic criteria for PTSD (Zoladz and Diamond, 2013). Reports indicate that lifetime prevalence of PTSD is twice as high in females as in males (Kilpatrick et al., 2013; Valentino et al., 2013a; Bangasser and Valentino, 2014).

Several studies document an association between PTSD and the development of an AUD and suggest that PTSD may precede the onset of AUD [reviewed in Gilpin and Weiner (2017)]. While the prevalence of AUD among patients with PTSD was estimated at 28% for women and 52% for men (Norman et al., 2012), data from the National Comorbidity Study indicated that 26.2% of women and 10.3% of men with alcohol dependence met the criteria for PTSD (Kessler et al., 1997). There also are data indicating that PTSD diagnosis more often preceded diagnosis of alcohol dependence in women than in men (Kessler et al., 1997; Sonne et al., 2003). Notably, in patients with comorbid PTSD/AUD, PTSD symptoms can promote excessive drinking, and alcohol abuse can worsen PTSD symptoms (Norman et al., 2012; Gilpin and Weiner, 2017). This reciprocal worsening of symptoms can negatively influence recovery outcomes and poor treatment response [see Albrechet-Souza and Gilpin (2019) and references therein]. Thus, the development of animal models can facilitate our understanding of neurobiological mechanisms contributing to comorbid PTSD/AUD and the advancement of effective pharmacological treatment strategies for this debilitating comorbidity.

We and others have found that exposure to predator stress (PS), which is considered a traumatic stress and used as a rodent model of PTSD (Dielenberg and McGregor, 2001; Cohen and Zohar, 2004; Matar et al., 2013; Deslauriers et al., 2018; Albrechet-Souza and Gilpin, 2019), significantly increased subsequent alcohol (ethanol) intake in male and female mice and rats, with evidence of heterogeneity in the response (Edwards et al., 2013; Manjoch et al., 2016; Finn et al., 2018; Ornelas et al., 2021). The heterogeneity that we have observed across multiple studies in male and female mice was consistent with results in rats following PS where an increase in ethanol intake or self-administration was observed only in animals characterized as exhibiting an “extreme behavioral response” (“EBR”; high anxiety) following PS (Manjoch et al., 2016), as “Avoiders” of a PS paired context (e.g., Edwards et al., 2013), or as exhibiting active coping behaviors during PS exposure (Ornelas et al., 2021). Additionally, various PS exposure [cat, soiled cat litter, dirty rat bedding, bobcat urine, TMT (2,5-dihydro-2,4,5-trimethylthiazoline)] significantly increased thermal nociception, startle reactivity, passive coping behaviors, and anxiety-related behaviors in male mice and rats (Belzung et al., 2001; Hebb et al., 2003; Cohen et al., 2008; Roltsch et al., 2014; Whitaker and Gilpin, 2015; Ornelas et al., 2021). Fewer comparable studies have been conducted in female rodents. However, recent work found that exposure to bobcat urine significantly increased startle reactivity in male but not in female rats (Albrechet-Souza et al., 2020), whereas exposure to TMT significantly increased passive coping behavior in female but not male rats (Ornelas et al., 2021), and exposure to rat, dirty rat bedding, or cat significantly increased anxiety-related behavior in both male and female mice (Toth et al., 2016; Finn et al., 2018; Shaw et al., 2020). Thus, it is possible that a high arousal, specific stress-reactive behaviors (e.g., passive coping), and/or anxiety state could contribute to the PS-induced increase in subsequent ethanol intake in a subgroup of animals.

Corticotropin releasing factor (CRF) in the paraventricular nucleus of the hypothalamus (PVN) controls the pituitary response to stress (Turnbull and Rivier, 1997). High levels of glucocorticoids exert negative feedback to shut off the hypothalamic-pituitary-adrenal (HPA) axis, but they also exert positive feedback at the level of the amygdala (Makino et al., 2002; Edwards et al., 2015; Finn, 2020). Acute administration of most drugs of abuse, including ethanol, activates the HPA axis, but these changes are blunted or dysregulated with repeated drug exposure (Koob, 2008; Richardson et al., 2008).

Abnormalities in responsivity of the HPA axis are observed in PTSD and AUD [reviewed in Norman et al. (2012) and Gilpin and Weiner (2017)]. For example, CRF peptide levels were significantly elevated following PS exposure in the central nucleus of the amygdala and ventromedial prefrontal cortex (vmPFC) of “Avoider” vs. “Non-Avoider” male rats, and subsequent studies determined that CRF-receptor 1 (CRF-R1) signaling in these brain regions mediated stress reactivity to PS and avoidance of stimuli paired with PS, respectively (Itoga et al., 2016; Schreiber et al., 2017). Exposure to PS also significantly increased plasma and fecal corticosterone (CORT) levels and plasma adrenocorticotropic hormone (ACTH) levels in male and female mice and rats (Mazor et al., 2009; Cozzoli et al., 2014; Whitaker and Gilpin, 2015; Finn et al., 2018; Albrechet-Souza et al., 2020; Shaw et al., 2020), with frequently greater increases in females vs. males (Cozzoli et al., 2014; Finn et al., 2018; Albrechet-Souza et al., 2020; Shaw et al., 2020). Consistent with sex differences in CRF-R1 signaling (Valentino et al., 2013a,b), we reported that a history of ethanol drinking and intermittent PS exposure produced sexually divergent changes in CRF-R1 protein levels in the hippocampus, with a significant elevation in CRF-R1 levels vs. naïve only in female mice (Finn et al., 2018). There also was a significant increase in glucocorticoid receptor (GR) levels in the medial prefrontal cortex (mPFC) of female but not male mice with a history of ethanol drinking and intermittent PS exposure vs. naïve (Finn et al., 2018). These previously observed changes in CRF-R1 and GR levels suggest that proteins regulating the HPA axis could be differentially affected by the history of ethanol drinking and trauma. On the other hand, this previous study included mild fluid restriction to entice higher ethanol consumption, which by itself could have affected expression of some of the components of the HPA axis.

Taken in conjunction with the above information, the purpose of the present set of studies was two-fold. One purpose was to begin to characterize “sensitivity” and “resilience” to PS-enhanced drinking in male and female mice in the absence of fluid restriction. Based on our observation of heterogeneity in the influence of intermittent PS on subsequent ethanol preference drinking (10% ethanol vs. water), we calculated median and interquartile ranges on all of our data in male and female C57BL/6J mice and then chose animals that were at or outside the interquartile range, resulting in two distinct subgroups and avoiding animals with values in the middle of the distribution. A similar strategy was utilized by Krishnan et al. (2007) to divide mice into “susceptible” and “unsusceptible” mice following repeated social defeat stress.

The second purpose was to determine whether PS-enhanced drinking in “sensitive” mice produced sex-dependent changes in levels of major proteins involved in the regulation of stress and anxiety responses. To build upon our prior work that measured CRF-R1 and GR levels in the mPFC and hippocampus, the present study utilized Western blots to quantify levels of CRF-R1, CRF-R2, CRF-binding protein (CRF-BP) and GR in mPFC and hippocampus. The mPFC and hippocampus are two brain regions important in the stress response that also exhibit plasticity in response to stress (e.g., Godsil et al., 2013; Bangasser and Valentino, 2014; McEwen et al., 2016; Morey et al., 2016; Zhu et al., 2016; Wang et al., 2018). Additionally, a projection originating in ventral hippocampus and terminating in mPFC is highly sensitive to stress (Godsil et al., 2013) and necessary for anxiety-related behavior (Padilla-Coreano et al., 2016).

## Materials and Methods

### Animals

**FIGURE 1:**
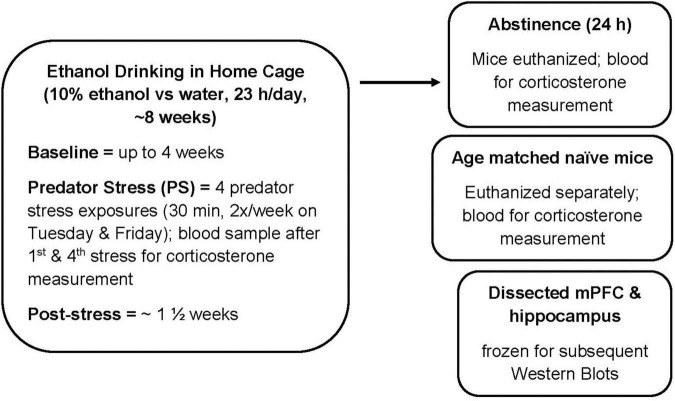
Experimental timeline. Male and female C57BL/6J mice were treated as described. Ethanol drinking (10% ethanol vs. water) was measured for 23 h per day in the home cage. Predator stress (PS) exposure occurred twice per week for 2 weeks, where mice were exposed to dirty rat bedding for 30 min. After the fourth PS exposure, post-stress drinking was measured for approximately 1 1/2 weeks. Mice were euthanized at 24 h after the final drinking session (and consumption of water for 24 h). For the Western Blot study, tissue from age-matched naïve mice was used as the comparator group. mPFC, medial prefrontal cortex.

C57BL/6J male and female mice were purchased from Jackson Laboratories West (Sacramento, CA, United States) at 8 weeks of age. Upon arrival, mice were acclimated to a reverse light/dark cycle (lights off at 1100) for up to 2 weeks. Rodent chow (Labdiet 5001 rodent diet; PMI international, Richmond, IN, United States) and water were freely available. Mice were individually housed with nestlets throughout the experiment. See experimental timeline (Figure 1) for details. Briefly, ethanol preference drinking (10% v/v ethanol vs. water; 23 h access) was measured for up to 8 weeks, with 2 weeks of intermittent PS exposure after baseline drinking. Mice were euthanized at 24 h after the final drinking session (and drinking water for 24 h). For the animals for which protein levels were measured with Western blots, dissected mPFC and hippocampus were frozen. Tissue also was harvested from age-matched naïve male and female C57BL/6J mice. The procedures were carried out in accordance with recommendations of the National Institute of Health *Guidelines for the Care and Use of Laboratory Animals* and were compliant with Institutional Animal Care and Use Committee approved protocols. All efforts were made to minimize distress and the number of animals used.

Studies characterizing PS-enhanced drinking were conducted in a total of 172 mice (117 males; 55 females). The Western Blot analysis was conducted on tissue from representative male and female animals in the “sensitive” and “resilient” subgroups and compared to tissue from age-matched naïve mice (10 females, 11 males). Separate groups of naïve mice were used as the comparator group for the plasma CORT analyses (21 females, 15 males).

### Predator Stress Exposure and Measurement of Plasma Corticosterone

Mice were removed from their home cage, transported to a different room, and placed into a polycarbonate cage containing dirty rat bedding for 30 min (Cozzoli et al., 2014; Finn et al., 2018). Mice were exposed to PS twice per week during the 2 weeks of intermittent PS exposure, and PS exposure occurred during the 1 h period prior to the next measurement of 23 h ethanol intake. Immediately after the first and fourth PS exposure, tail blood samples (∼20 μL) were collected rapidly for subsequent measurement of plasma CORT levels *via* radioimmunoassay and using a commercially available kit (ImmunoChem Double Antibody Corticosterone for rodents; MP Biomedicals, Santa Ana, CA, United States; Cozzoli et al., 2014; Finn et al., 2018). Upon euthanasia, trunk blood was collected from the experimental mice to assess plasma CORT levels after consuming ethanol with intermittent PS and a 24 h period of abstinence. Trunk blood also was collected upon euthanasia from a separate group of naïve mice for baseline CORT levels. The naïve mice were not moved to a novel (clean) cage prior to the blood sampling.

### Identification of “Sensitive” and “Resilient” Subgroups

Based on our prior work (Finn et al., 2018) where ethanol intake increased following exposures to PS2-4, we averaged ethanol intake following PS2-4. Baseline (BL) drinking was averaged over days of stable ethanol drinking prior to the first PS exposure, and typically ranged from 1 to 3 weeks. Then, the PS-induced change in ethanol intake was calculated for each animal as the percent (%) change in intake (average PS2-4) from BL. We calculated median and interquartile ranges on the % change in drinking and then chose animals that were at or outside the interquartile range, resulting in two distinct subgroups and avoiding animals with values in the middle of the distribution. Additionally, for mice to be placed in the “PS-sensitive” or “sensitive” subgroup, there had to be ≥20% increase in averaged ethanol intake following PS2-4 vs. BL. For mice in the “PS-resilient” or “resilient” subgroup, ethanol intake was unchanged or decreased vs. BL. This criterion established the basis for the “sensitive” and “resilient” subgroups.

### Assessment of Select Protein Levels Using Western Blot Analysis

Immunoblotting was performed on mPFC (including prelimbic, cingulate, and overlying motor cortex) and entire hippocampal tissues that were dissected freehand from mouse brains chilled on ice. These tissues were obtained from animals in the “sensitive” and “resilient” subgroups and from age-matched naïve mice. All samples were placed in microcentrifuge tubes (1.5 mL), frozen immediately on dry ice, and stored at −80°C.

Protein isolation and immunoblotting were conducted according to routine procedures in the Devaud lab (Finn et al., 2018; Devaud et al., 2020), with slight modification. We used a method of tissue homogenization to maximize the amount of tissue from the brain regions and to allow us to make comparisons across a number of proteins rather than focusing on mPFC and hippocampal subregions. Briefly, tissue was homogenized using an electric homogenizer. Lysates were prepared using RIPA buffer (Cat# ab156034; Abcam; Cambridge, MA, United States), and a total particulate fraction was collected by centrifugation (20 min at 12,000 rpm at 4°C). Protein concentrations for each tissue were determined using the Thermo Scientific BCA Assay kit (Pierce™ Cat# 23227; Rockport, IL, United States). Sample homogenates (20 μg protein/sample) were diluted with sample loading buffer (Bio-Rad, Hercules, CA, United States), denatured for 3 min, and then separated using a 10-well 4–15% Tris-glycine gel in a Bio-Rad Western Blot apparatus. Each gel had 3 naïve, 3 “sensitive” subgroup, and 3 “resilient” subgroup samples from the same tissue and sex for within blot comparisons. After separation, proteins were transferred to PVDF membranes (Bio-Rad Immun-Blot^®^ PVDF).

Blots were incubated with selected antibodies: CRF-R1 (1:500; Cat# PA5-27121, Thermo Fisher Scientific), CRF-R2 (1:500; Cat# PA5-23129, Thermo Fisher Scientific), CRF-BP (1:250; Cat# PA5-61157, Thermo Fisher Scientific), and GR (1:200; Cat# PA1-512, Thermo Fisher Scientific). We used β-actin (1:500; Cat#: 47778, Santa Cruz Biotechnology, Dallas, TX, United States) as the loading control based on our earlier results showing that β-actin peptide levels did not change after chronic ethanol exposure and/or withdrawal (Devaud et al., 1997). After incubation with the primary antibody, blots were washed and incubated with the appropriate HRP-conjugated secondary antibody (1:8000). Blots were then incubated in chemiluminescent substrate (HyGlo, Denville Sci, Holliston, MA, United States), and signals were captured using a Bio-Rad ChemiDoc MP imager. Representative immunoblots for each protein, brain region, and sex are depicted in Supplementary Figures 1–4. Some blots showed several bands, reflecting alternative isoforms or previous immunoreactions. The proteins of interest were identified, based on size, using Precision Plus Protein™ WesternC™ Blotting Standard Ladders (Bio-Rad). Relative density measurements of bands corresponding to the main product of the proteins were collected and quantified using the Image Lab software.

### Statistical Analysis

**FIGURE 2:**
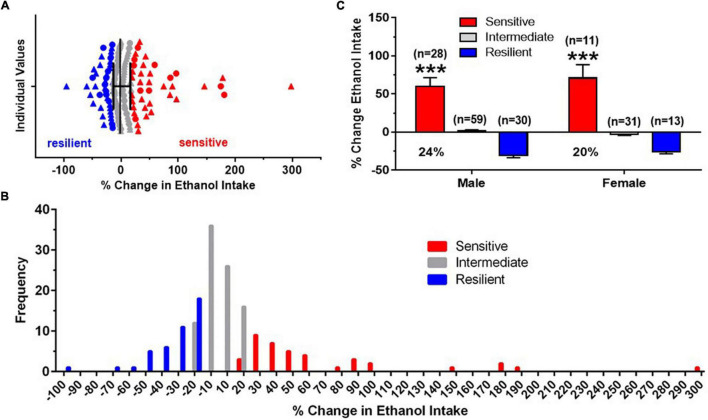
Identification of “sensitive” and “resilient” subgroups. **(A)** Horizontal scatterplot of the distribution of percent (%) change in baseline (BL) ethanol drinking after four predator stress (PS) exposures, with median and interquartile ranges for male (triangles) and female (circles) C57BL/6J mice. Mice chosen for the “sensitive” (red) and “resilient” (blue) subgroups were the extremes of the distribution and did not include animals in the middle (gray, “intermediate”). **(B)** Frequency distribution histogram for the % change in BL ethanol drinking after intermittent PS further illustrate the distinct “sensitive” and “resilient” subgroups. **(C)** Mean ± SEM % change in ethanol intake data for the three subgroups, with the number of animals in each subgroup shown in parentheses. The proportion of mice in the “sensitive” subgroup is shown below the mean data for this group. Data for “intermediate” group are shown for informational purposes. Analyses were conducted across the “sensitive” and “resilient” subgroups. ***p < 0.001 vs. resilient subgroup.

Although males and females were tested in separate cohorts, the drinking data, the Western blot data, and the CORT data were analyzed with sex as a factor in the ANOVAs. *Post-hoc* tests utilized ANOVA with Tukey’s *post-hoc* tests or between group *t*-tests. For the scatterplots, individual values for the males and females combined were analyzed in Excel to calculate the median and interquartile ranges. Pearson’s correlations between final CORT levels and the Western blot data were conducted in Prism. The remainder of the data are presented as mean ± SEM, and analyses were conducted with SYSTAT (version 13, SYSTAT Software Inc., Richmond, CA, United States). Data from animals in the “sensitive” and “resilient” subgroups were analyzed (vs. naïve, as appropriate); data in the “intermediate” subgroup were not included in the analyses and are depicted in Figure 2 for informational purposes. The level of significance was set at *p* ≤ 0.05. Graphs were prepared in Prism (version 6, GraphPad Software, San Diego, CA, United States).

For the drinking data, the dependent variables were daily ethanol intake (g/kg; 10% ethanol solution), ethanol preference (volume of 10% ethanol/volume of total fluid), average BL intake (g/kg), average intake after PS2-4 (g/kg), % change in ethanol intake (average PS2-4 minus average BL/average BL × 100), and plasma CORT levels. Ethanol intake and preference also were averaged into five blocks: BL, PS1 (after first PS, 2 days), PS2 (after second PS, 3 days), PS3 (after third PS, 2 days), and PS4 (after fourth PS, 3 days). Depending on significance of interaction, follow-up ANOVAs and Tukey’s *post-hoc* tests examined the effect of PS in each subgroup or examined the effect of subgroup in each sex. For the CORT data, we were not able to examine subgroup vs. naïve statistically, since there was only one naïve group per sex.

For the protein data, measurements were normalized to the β-actin signal for equivalent protein loading. Three to five data points were collected from each treatment subgroup animal across independent immunoblots, converted to % of naïve values, and then the converted values were averaged for each animal for summary and analyses. Western blots were conducted on tissue from each brain region in separate analyses; each gel had 3 naïve, 3 “sensitive” subgroup, and 3 “resilient” subgroup samples from the same tissue and sex for within blot comparisons. Thus, these data were normalized to the respective naïve group for each sex and brain region. Then Western blot data for each brain region were analyzed for sex and subgroup effects using two way ANOVA, with follow-up one way ANOVA with Tukey’s *post-hoc* tests.

## Results

### Initial Characterization of “Sensitive” and “Resilient” Subgroups

By analyzing a large number of animals for each sex, median and interquartile ranges were calculated on the individual data (% change in average PS2-4 intake from BL). A scatterplot of these data, combined across sex, is depicted in Figure 2A. These data illustrate that animals chosen for the “sensitive” and “resilient” subgroups represented distinct groups of mice and did not include animals with values in the middle of the distribution. Additionally, mice in the “sensitive” subgroup exhibited a minimum of a 20% increase in ethanol intake. A frequency distribution histogram (Figure 2B) also revealed a segregation of “sensitive” mice from “resilient” mice and provided additional support for the validity of distinct “sensitive” and “resilient” subgroups. Across all the mice tested, ethanol intake was significantly increased in the “sensitive” subgroup over BL by 60% in males and by 71% in females, with 24% of the males and 20% of the females being categorized as “sensitive” (Figure 2C). The two way ANOVA revealed that there was no significant effect of sex, a significant effect of subgroup [*F*(1,78) = 81.01, *p* < 0.001], and no interaction. The similar and significant subgroup differences in drinking in males and females reflected our use of identical criteria for defining “sensitive” and “resilient” animals across males and females.

**FIGURE 3:**
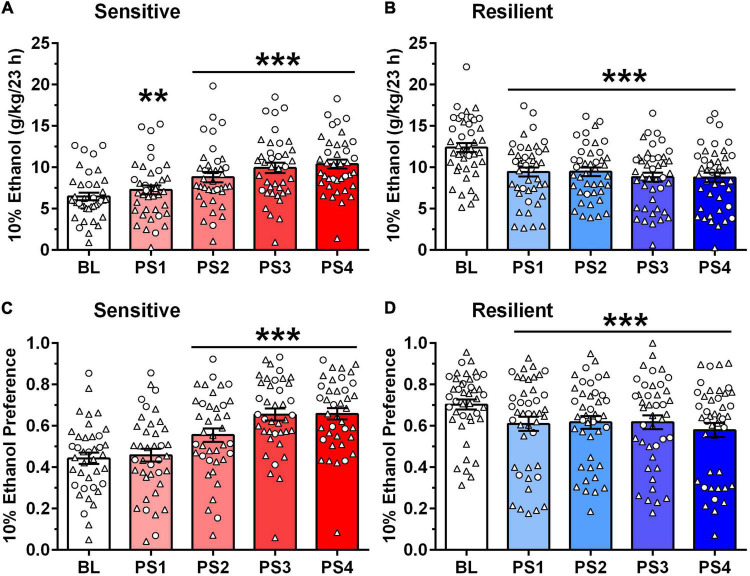
Different pattern of ethanol intake **(A,B)** and preference **(C,D)** after intermittent predator stress (PS) in “sensitive” vs. “resilient” subgroups. Depicted are mean ± SEM intake of 10% ethanol (g/kg/23 h) intake **(A,B)** and 10% ethanol preference **(C,D)** for the “sensitive” and “resilient” animals depicted in Figure 2. Individual data points are shown for the sexes combined (males = triangles; females = circles). Ethanol intake and preference were averaged into five blocks: baseline (BL), PS1 (after first PS, 2 days), PS2 (after second PS, 3 days), PS3 (after third PS, 2 days), and PS4 (after fourth PS, 3 days). **(A,B)** Ethanol intake began increasing significantly vs. BL after PS1 in the “sensitive” subgroup, whereas ethanol intake decreased significantly vs. BL after PS1-4 in the “resilient” subgroup. Following PS4, ethanol intake increased over BL by 62% (males) and 59% (females) in the “sensitive” subgroup, and decreased from BL by 30% (males) and 27% (females) in the “resilient” subgroup. **(C,D)** Ethanol preference increased significantly vs. BL after PS2-4 in the “sensitive” subgroup, whereas ethanol preference decreased significantly vs. BL after PS1-4 in the “resilient” subgroup. Following PS4, ethanol preference increased over BL by 44% (males) and 61% (females) in the “sensitive” subgroup, and decreased from BL by 18% (males) and 16% (females) in the “resilient” subgroup. **p < 0.01, ***p ≤ 0.001 vs. respective BL.

Furthermore, a 20% increase in 23 h ethanol intake in the “sensitive” subgroups reflected greater than a 2 g/kg increase over BL in the male and female mice (Figure 3A). For these follow-up analyses, ethanol intake was averaged into five blocks: BL, PS1 (after first PS, 2 days), PS2 (after second PS, 3 days), PS3 (after third PS, 2 days), and PS4 (after fourth PS, 3 days). The initial analysis revealed that ethanol intake was significantly higher in females vs. males, tended to be influenced by subgroup, and significantly influenced by PS exposure [Sex: *F*(1,76) = 38.36, *p* < 0.001; Subgroup: *F*(1,76) = 3.44, *p* < 0.07; PS: *F*(4,304) = 8.32, *p* = 0.006]. The interaction between subgroup and PS exposure also was significant [*F*(4,304) = 67.20, *p* < 0.001], but no interactions with sex were statistically significant. When collapsed across sex, there was a trend for an effect of subgroup [*F*(1,78) = 3.19, *p* < 0.08], significant effect of PS [*F*(4,312) = 8.31, *p* < 0.001] and significant subgroup by PS interaction [*F*(4,312) = 68.04, *p* < 0.001]. In the “sensitive” subgroup, PS exposure significantly influenced ethanol intake [*F*(4,148) = 38.84, *p* < 0.001], with *post-hoc* tests confirming that ethanol intake was increased significantly over BL following PS1-4 (Figure 3A). Ethanol intake increased by 31% (males) and 44% (females) over BL after PS2 and further increased to 62% (males) and 59% (females) over BL after PS4. Ethanol intake after PS2–4 also was significantly higher than intake following PS1 (*p*s ≤ 0.01; not depicted on figure). However, in the “resilient” subgroup, PS exposure significantly altered ethanol intake [*F*(4,164) = 36.94, *p* < 0.001], with *post-hoc* tests confirming a significant decrease in ethanol intake following PS1-4 vs. BL (Figure 3B). Following PS2, ethanol intake decreased by 22–25% from BL, whereas ethanol intake decreased by 27–30% from BL after PS4, in the female and male mice, respectively.

Baseline ethanol intake (see Figures 3A,B) was higher in females vs. males [*F*(1,78) = 31.47, *p* < 0.001] and also differed across subgroups [*F*(1,78) = 87.75, *p* < 0.001]. There was no significant interaction between sex and subgroup. When collapsed across sex, a follow-up *t*-test indicated that BL intake in the “sensitive” subgroup was significantly lower than intake in the “resilient” subgroup [*t*(80) = 8.17, *p* < 0.001; not shown on figure]. However, repeated exposure to PS reversed this relationship, such that after PS4, ethanol intake was significantly higher in the “sensitive” vs. “resilient” subgroup (*p* < 0.05; not shown on figure).

To test whether PS specifically affected ethanol intake, or non-specifically affected consummatory behaviors, similar analyses were performed for ethanol preference (Figures 3C,D). The initial analysis revealed that ethanol preference tended to be higher in females vs. males and was significantly influenced by subgroup and PS exposure [Sex: *F*(1,77) = 3.66, *p* < 0.06; Subgroup: *F*(1,77) = 4.14, *p* < 0.05; PS: *F*(4,308) = 13.00, *p* < 0.001]. The interaction between subgroup and PS exposure also was significant [*F*(4,308) = 30.72, *p* < 0.001], but no interactions with sex were statistically significant. When collapsed across sex, ANOVA revealed a trend for an effect of subgroup [*F*(1,79) = 3.58, *p* = 0.06], a significant effect of PS [*F*(4,316) = 14.14, *p* < 0.001] and significant subgroup by PS interaction [*F*(4,316) = 34.26, *p* < 0.001]. In the “sensitive” subgroup, PS exposure significantly influenced ethanol preference [*F*(4,152) = 36.37, *p* < 0.001], with *post-hoc* tests confirming that ethanol preference was increased significantly over BL following PS2–4 (Figure 3C). Ethanol preference increased by 42–44% (males) and 61–62% (females) over BL after PS3 and PS4, respectively. Ethanol preference also was significantly higher following PS2–4 vs. preference following PS1 (*p*s < 0.001; not shown on figure). In the “resilient” subgroup (Figure 3D), PS exposure significantly altered ethanol preference [*F*(4,164) = 8.17, *p* < 0.001], with *post-hoc* tests indicating that preference was decreased significantly from BL following PS1-4. Thus, the effects of PS on ethanol preference were similar to the effects on ethanol intake.

Baseline ethanol preference (see Figures 3C,D) did not differ in females vs. males, but it was significantly affected by subgroup [*F*(1,78) = 49.46, *p* < 0.001]. There was no significant interaction between sex and subgroup. When collapsed across sex, a follow-up *t*-test indicated that BL preference in the “sensitive” subgroup was significantly lower than in the “resilient” subgroup [*t*(80) = 7.09, *p* < 0.001; not shown on figure]. However, repeated exposure to PS eliminated this difference, such that preference tended to be higher at PS4 (*p* = 0.08) in the “sensitive” vs. “resilient” subgroups.

**FIGURE 4:**
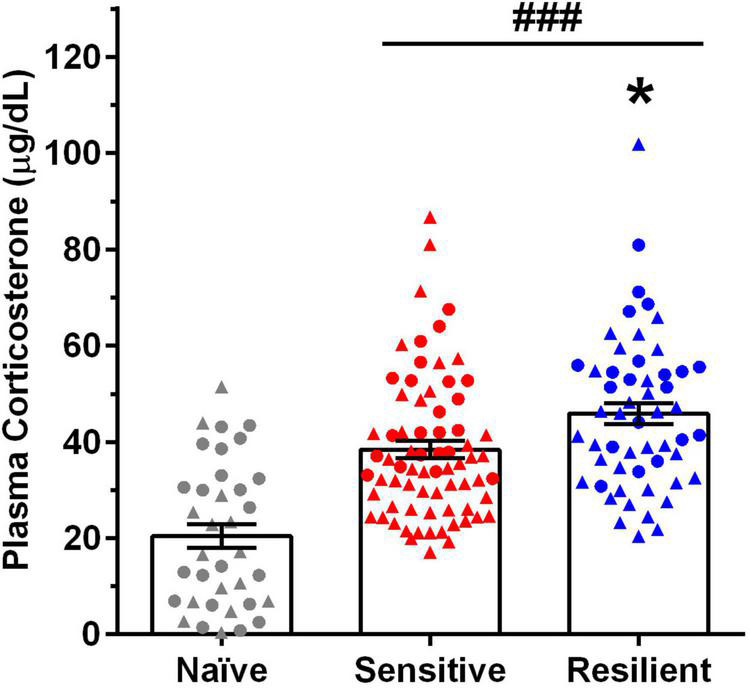
Predator stress (PS) significantly increases plasma corticosterone (CORT) levels, with greater increases in the “resilient” vs. “sensitive” subgroup. Depicted are mean ± SEM plasma CORT levels for male and female mice following exposure to the first and fourth predator stress (PS1 and PS4) vs. values in naïve mice. Individual data points are shown collapsed across sex (males = triangles, females = circles) and PS. Group size: naïve (15 male, 21 female), “sensitive” subgroup (25 male, 11 female per PS), “resilient” subgroup (17 males, 10 females per PS). Plasma CORT levels increased significantly over naïve values following PS, and they were significantly higher in the “resilient” (↑124%) subgroup vs. values in the “sensitive” (↑88%) subgroup. *p < 0.05 vs. “sensitive” subgroup; ^###^*p* < 0.001 vs. naïve.

Plasma CORT levels for naïve mice and experimental mice in the “sensitive” and “resilient” subgroups following exposure to PS1 and PS4 are summarized in Figure 4. The initial analysis revealed that plasma CORT levels were higher in females vs. males and were increased significantly following PS [Sex: *F*(1,152) = 13.42, *p* < 0.001; PS: *F*(4,152) = 18.58, *p* < 0.001], but the interaction between sex and PS was not significant. When data were collapsed across sex, including the naïve group, and collapsed across PS1 and PS4, a one way ANOVA and *post-hoc* tests confirmed that plasma CORT levels were significantly increased by PS [*F*(2,159) = 30.73, *p* < 0.001] vs. naïve and were significantly higher in the “resilient” vs. the “sensitive” subgroup (Figure 4). The PS-induced increase in plasma CORT levels over naïve was 124% for animals in the “resilient” subgroup, when compared with the 88% increase in the “sensitive” subgroup.

Plasma CORT levels following a 24 h abstinence after the final drinking session in the “sensitive” and “resilient” subgroups (i.e., upon euthanasia) are shown in Table 1. The initial analysis, which included the naïve group, indicated that final CORT levels were significantly higher in females vs. males and altered by subgroup [Sex: *F*(1,98) = 47.56, *p* < 0.001; Subgroup: *F*(2,98) = 15.65, *p* < 0.001]. The interaction between sex and subgroup also was significant [*F*(2,98) = 9.21, *p* < 0.001]. In male mice, final CORT levels were not significantly altered vs. naïve in the “sensitive” and “resilient” subgroups. In contrast, final CORT levels in females were significantly different [*F*(2,42) = 14.44, *p* < 0.001], with *post-hoc* tests confirming that CORT levels were significantly elevated vs. naïve in both the “sensitive” and “resilient” subgroups (*ps* < 0.001).

**TABLE 1:** Final plasma corticosterone levels.

	Naïve	“Sensitive” subgroup	“Resilient” subgroup
Male	18.101 ± 3.849 (*n* = 15)	23.415 ± 2.453 (*n* = 27)	23.117 ± 2.958 (*n* = 17)
Female	22.107 ± 3.308 (*n* = 21)	63.963 ± 11.498*** (*n* = 11)	57.705 ± 5.827*** (*n* = 13)

Shown are mean ± SEM plasma corticosterone levels (μg/dL) following a 24 h abstinence after the final drinking session in the “sensitive” and “resilient” subgroups (i.e., upon euthanasia) and in separate groups of naïve animals. Values for the naïve group, collapsed across sex, also are depicted in Figure 4. ***p < 0.001 vs. respective naïve group.

### Sex- and Subgroup-Dependent Changes in Protein Levels Related to Stress-Regulating Systems

Western blots were conducted on tissue from representative animals in the “sensitive” and “resilient” subgroups that are depicted in Figures 2, 3, and protein levels were compared to levels in tissue from age-matched naïve controls. For this cohort of animals in the “sensitive” and “resilient” subgroups, the % change in ethanol intake from BL was comparable to the results depicted in Figure 2C; ethanol drinking was increased 69% in the males and 46% in the females. Differences in BL ethanol intake between the “sensitive” and “resilient” subgroups also were comparable to the results depicted in Figures 3A,B, with significantly lower BL ethanol intake in the mice from the “sensitive” vs. “resilient” subgroup. Plasma CORT levels were comparable to the results depicted in Figure 4 and Table 1. Plasma CORT levels following intermittent PS were significantly higher in the experimental groups vs. naïve and were significantly higher in the “resilient” vs. “sensitive” subgroup (Figure 4). Additionally, final plasma CORT levels remained elevated over naïve in the “sensitive” and “resilient” subgroups only in the female mice (Table 1).

**FIGURE 5:**
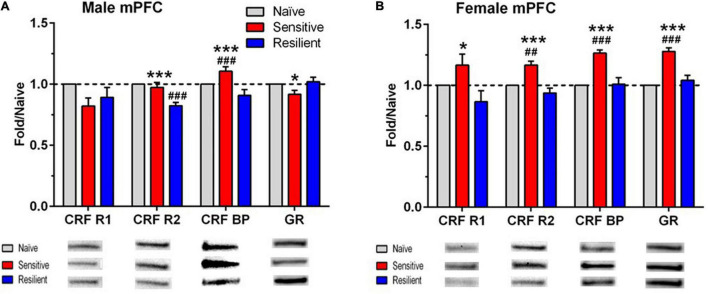
Sex- and subgroup-dependent changes in protein levels related to stress-regulating systems in the medial prefrontal cortex (mPFC) after a history of ethanol drinking and intermittent predator stress exposure. There were divergent, treatment- and subgroup-induced alterations in the proteins examined, with sex differences observed for CRF-R1, CRF-R2, and GR. Representative animals from the “sensitive” and “resilient” subgroups depicted in Figures 2, 3 were chosen for the Western blot analyses, and values were compared to separate groups of naïve mice. Values are mean ± SEM levels that were normalized to β-actin and then normalized to the respective naïve group (dashed line) for male **(A)** and female **(B)** mice. Changes in relative protein levels were compared using normalized optical density measurements. Representative bands from the immunoblots for each protein are included beneath each panel on the graph. Representative immunoblots for each protein, brain region, and sex are depicted in Supplementary Figures 1–4. Group size: naïve (10–11 male, 8–10 female), “sensitive” subgroup (7 male, 5 female), “resilient” subgroup (10 males, 8 females). *p ≤ 0.05, ***p ≤ 0.001 vs. respective “resilient” subgroup; ^##^*p* ≤ 0.01, ^###^*p* ≤ 0.001 vs. respective naïve. The results depicted reflect either separate analyses for each sex (CRF R1, CRF R2, GR) or analyses with the sexes combined (CRF BP). See Table 2 for ANOVAs. CRF R1, corticotropin releasing factor receptor 1; CRF R2, corticotropin releasing factor receptor 2; CRF BP, corticotropin releasing factor binding protein; GR, glucocorticoid receptor.

**FIGURE 6:**
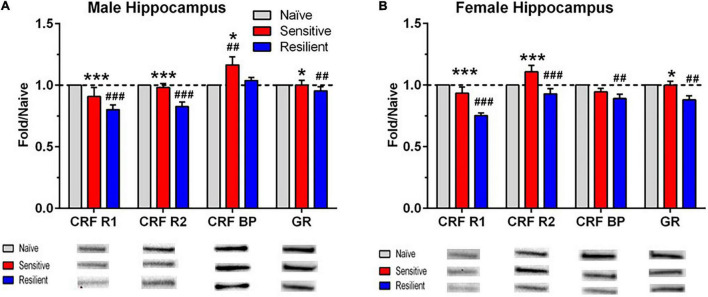
Sex- and subgroup-dependent changes in protein levels related to stress-regulating systems in the hippocampus after a history of ethanol drinking and intermittent predator stress exposure. There were treatment- and subgroup-induced alterations in the proteins examined, with sex differences only observed for CRF-BP. Representative animals from the “sensitive” and “resilient” subgroups depicted in Figures 2, 3 were chosen for the Western blot analyses, and values were compared to separate groups of naïve mice. Values are mean ± SEM levels that were normalized to β-actin and then normalized to the respective naïve group (dashed line) for male **(A)** and female **(B)** mice. Changes in relative protein levels were compared using normalized optical density measurements. Representative bands from the immunoblots for each protein are included beneath each panel on the graph. Representative immunoblots for each protein, brain region, and sex are depicted in Supplementary Figures 1–4. Group size is the same as in Figure 5. *p ≤ 0.05, ***p ≤ 0.001 vs. respective “resilient” subgroup; ^##^*p* ≤ 0.01, ^###^*p* ≤ 0.001 vs. respective naïve. The results depicted reflect either separate analyses for each sex (CRF BP) or analyses with the sexes combined (CRF R1, CRF R2, GR). See Table 2 for ANOVAs. CRF R1, corticotropin releasing factor receptor 1; CRF R2, corticotropin releasing factor receptor 2; CRF BP, corticotropin releasing factor binding protein; GR, glucocorticoid receptor.

The Western blot results are depicted in Figures 5, 6, with representative images for each protein shown in Supplementary Figures 1–4. The corresponding ANOVA results are shown in Table 2. The proteins examined were CRF-R1, CRF-R2, CRF-BP, and GR. Significant interactions between sex and subgroup documented that there were sexually divergent changes in the “sensitive” subgroup for protein levels of CRF-R1, CRF-R2, and GR in the mPFC. In the female mPFC (Figure 5B), all four proteins examined were significantly elevated in the “sensitive” vs. “resilient” subgroups, and the proteins were also increased vs. the naïve group by 17–28%; the increase vs. naïve was significant for all proteins except for CRF-R1. In contrast, levels of the four proteins in male mPFC (Figure 5A) were not elevated significantly in the “sensitive” subgroup vs. the naïve group, and there was a significant decrease in levels of CRF-R2 in the “resilient” subgroup vs. naïve. Since CRF-BP did not show a significant sex by subgroup interaction, data on this protein were collapsed across sex. One way ANOVA and subsequent Tukey’s *post-hoc* test showed a significant 17% increase in levels of this protein in “sensitive” vs. naïve mice and 22% higher levels in “sensitive” vs. “resilient” subgroups.

**TABLE 2:** ANOVAs for Western Blot analyses.

	CRF-R1	CRF-R2	CRF-BP	GR
**mPFC**				
Main effect: Sex	*F*(1,42) = 3.81, *p* = 0.058	*F*(1,42) = 20.85, *p* < 0.001	*F*(1,42) = 8.24, *p* = 0.006	*F*(1,42) = 27.86, *p* < 0.001
Main effect: Subgroup	*F*(2,42) = 2.34, *p* = 0.108	*F*(2,42) = 24.15, *p* < 0.001	*F*(2,42) = 18.91, *p* < 0.001	*F*(2,42) = 5.08, *p* = 0.01
Interaction	*F*(2,42) = 4.24, *p* = 0.02	*F*(2,42) = 6.08, *p* = 0.005	*F*(2,42) = 2.35, *p* = 0.108	*F*(2,42) = 20.30, *p* < 0.001
Male	*F*(2,24) = 2.16, *p* = 0.137	*F*(2,24) = 16.54, *p* < 0.001		*F*(2,24) = 3.35, *p* = 0.05
Female	*F*(2,18) = 3.99, *p* = 0.037	*F*(2,18) = 13.26, *p* < 0.001		*F*(2,18) = 20.89, *p* < 0.001
Sex combined			*F*(2,45) = 14.93, *p* < 0.001	
**Hippocampus**				
Main effect: Sex	*F*(1,45) = 0.073, *p* = 0.788	*F*(1,45) = 9.94, *p* = 0.003	*F*(1,45) = 23.84, *p* < 0.001	*F*(1,46) = 1.50, *p* = 0.227
Main effect: Subgroup	*F*(2,45) = 25.45, *p* < 0.001	*F*(2,45) = 16.91, *p* < 0.001	*F*(2,45) = 4.03, *p* = 0.025	*F*(2,46) = 7.20, *p* = 0.002
Interaction	*F*(2,45) = 0.568, *p* = 0.571	*F*(2,45) = 2.83, *p* = 0.069	*F*(2,45) = 6.70, *p* = 0.002	*F*(2,46) = 1.38, *p* = 0.261
Male			*F*(2,25) = 6.24, *p* = 0.006	
Female			*F*(2,20) = 6.02, *p* = 0.009	
Sex combined	*F*(2,48) = 25.89, *p* < 0.001	*F*(2,48) = 14.10, *p* < 0.001		*F*(2,49) = 6.43, *p* = 0.003

*Shown are the results from the two way ANOVAs (sex, subgroup) and follow-up one way ANOVAs that were conducted across the data from naïve, “sensitive” and “resilient” animals for each protein and brain region, and that correspond to the results depicted in Figures 5, 6. CRF-R1, corticotropin releasing factor receptor 1; CRF-R2, corticotropin releasing factor receptor 2; CRF-BP, corticotropin releasing factor binding protein; GR, glucocorticoid receptor; mPFC, medial prefrontal cortex.*

While significant effects of subgroup were found across all four investigated proteins in the hippocampus, interactions between sex and subgroup reached statistical significance only for CRF-BP (Figures 6A,B). Therefore, data were collapsed across sex for CFR-R1, CRF-R2, and GR and analyzed. CRF-R1 levels were decreased significantly in the “resilient” subgroup vs. naïve by 22% and vs. “sensitive” mice by 14%. In the same direction of effects, CRF-R2 levels were decreased significantly in the “resilient” subgroup vs. naïve by 13% and vs. “sensitive” mice by 16%. Also matching this direction, albeit with weaker effects, GR levels were decreased significantly in the “resilient” subgroup vs. both naïve and “sensitive” mice by 8%. Reflecting the significant interaction of subgroup with sex for CRF-BP, its hippocampal levels were significantly higher in the “sensitive” subgroup by 17% vs. naïve and also higher than in the “resilient” group (Figure 6A) in male, but not female subjects. In contrast, female hippocampal CRF-BP levels were significantly lower in “resilient” mice vs. naïve by 11% (Figure 6B). Overall, “sensitivity” and “resilience” to PS-enhanced drinking produced sexually divergent changes in levels of several proteins important for stress regulation that also varied with the brain region examined.

Due to the significantly higher CORT levels upon euthanasia in the female vs. male experimental mice, correlations between final CORT levels and stress-related protein levels were conducted (Table 3). In the mPFC, final CORT levels were significantly, positively correlated with CRF-BP and GR levels, and there was a strong trend for a positive correlation with CRF-R1 levels, only in the “sensitive” subgroup. CRF-R2 levels were significantly, positively correlated with final CORT levels in the “resilient” subgroup, with a weak trend for a similar relationship in the “sensitive” subgroup. In the hippocampus, fewer significant correlations were observed. In the “resilient” subgroup, final CORT levels were significantly, positively correlated with CRF-R2 levels and significantly, negatively correlated with CRF-BP levels.

**TABLE 3:** Correlations between stress-related proteins and final plasma corticosterone levels.

Brain region	Subgroup	Variable	CRF-R1	CRF-R2	CRF-BP	GR
mPFC	“Sensitive”	Final CORT (μg/dL)	*r* = 0.56	*r* = 0.49	***r* = 0.70**	***r* = 0.69**
			*p* < 0.06	*p* < 0.11	***p* = 0.01**	***p* = 0.01**
			*n* = 12	*n* = 12	*n* = 12	*n* = 12
	“Resilient”	Final CORT (μg/dL)	*r* = −0.29	***r* = 0.48**	*r* = 0.25	*r* = −0.02
			*p* = 0.25	***p* < 0.05**	*p* = 0.31	*p* = 0.93
			*n* = 18	*n* = 18	*n* = 18	*n* = 18
Hippocampus	“Sensitive”	Final CORT (μg/dL)	*r* = 0.12	*r* = 0.43	*r* = −0.38	*r* = 0.18
			*p* = 0.71	*p* = 0.16	*p* = 0.22	*p* = 0.57
			*n* = 12	*n* = 12	*n* = 12	*n* = 12
	“Resilient”	Final CORT (μg/dL)	*r* = −0.35	***r* = 0.49**	***r* = **−**0.50**	*r* = −0.32
			*p* = 0.16	***p* < 0.05**	***p* < 0.05**	*p* = 0.20
			*n* = 18	*n* = 18	*n* = 18	*n* = 18

*Final corticosterone (CORT) levels were obtained from experimental animals following a 24 h abstinence after the final drinking session in the “Sensitive” and “Resilient” subgroups (i.e., upon euthanasia). Pearson’s correlations were conducted between final CORT levels and levels of the stress-related proteins, on data that were collapsed across sex. Significant correlations are depicted in bold font.*

*mPFC, medial prefrontal cortex; CRF-R1, corticotropin releasing factor receptor 1; CRF-R2, corticotropin releasing factor receptor 2; CRF-BP, corticotropin releasing factor binding protein; GR, glucocorticoid receptor.*

## Discussion

The purpose of the present studies was to begin to characterize “sensitivity” and “resilience” to PS-enhanced drinking in male and female C57BL/6J mice. These studies build upon our prior work, where we determined that repeated exposure to intermittent PS (30 min dirty rat bedding) significantly increased anxiety-related behavior and plasma CORT levels (Cozzoli et al., 2014; Finn et al., 2018) as well as neuronal activation in the hippocampus and mPFC (Finn and Ryabinin, unpublished) in naïve male and female C57BL/6J mice. We and others also found that exposure to PS significantly increased subsequent ethanol intake in male and female mice and rats, with evidence of heterogeneity in the response (Edwards et al., 2013; Manjoch et al., 2016; Finn et al., 2018; Ornelas et al., 2021). For our work in male and female mice, we chose a strategy similar to that employed by others (e.g., Krishnan et al., 2007) whereby animals at the extremes of a Gaussian distribution were chosen, based on the change in 10% ethanol intake from BL after intermittent PS. This resulted in two distinct subgroups of “sensitive” and “resilient” animals to PS-enhanced drinking, and the criterion can be utilized to examine mechanisms contributing to “sensitivity” and “resilience” to PS-enhanced drinking. Cohen et al. (2014) also use a retrospective analysis of behavioral data to classify animals as being severely, partially, or minimally affected by PS, based on behavioral criteria. Ornelas et al. (2021) examined individual differences in stress-reactive behaviors during PS (e.g., digging and immobility) and determined that rats engaging in more active coping behaviors (high digging/low immobility ratio scores) also exhibited a persistent increase in ethanol self-administration. Advantages of this strategy are that it allows for the correlation of specific molecular or physiological outcomes with the degree and pattern (or subgroup) of individual behavioral responses as well as for the calculation of prevalence rate as a study parameter (Cohen et al., 2014). Notably, the proportion of animals characterized as “PS-sensitive” in our studies (<25%) is comparable to reports on prevalence of PTSD and AUD in men and women (Kessler et al., 1997; Sonne et al., 2003; Norman et al., 2012; Gilpin and Weiner, 2017).

The analysis of drinking following each PS exposure revealed that ethanol intake was increased significantly over BL after PS2–4 in both males and females from the “sensitive” subgroup. There also were comparable increases in ethanol drinking over BL in the sexes; ethanol intake increased over BL by 31% (males) and 44% (females) after PS2 and by 62% (males) and 59% (females) after PS4. The increases in ethanol intake over BL after PS2 in the “sensitive” subgroup, using a 23 h access procedure, are comparable with our earlier work that utilized a 2 h limited access procedure and found that 2 h 10% ethanol intake was increased by 30–35% in males and by 25–36% in females on the second day after PS exposure (Cozzoli et al., 2014). One other laboratory has shown that PS exposure increases ethanol intake in female rats, and in this study, the persistent increase in ethanol self-administration was associated with more active coping behaviors during TMT exposure; ethanol self-administration was not increased in male or female rats that exhibited passive coping behaviors during TMT exposure (Ornelas et al., 2021). Notably, all studies in rats showing PS-enhanced drinking administered the PS after a period of baseline ethanol intake (Edwards et al., 2013; Roltsch et al., 2014; Manjoch et al., 2016; Ornelas et al., 2021), consistent with our procedure. Thus, the establishment of baseline ethanol intake prior to PS exposure appears crucial to the subsequent determination of PS-enhanced drinking in a proportion of male and female rodents.

In the current and prior study that measured 23 h drinking, we found that the significant escalation in ethanol intake began after exposure to the second PS. This timing corresponded to the significant increase in anxiety-related behavior that was observed after exposure to the second PS in naïve male and female mice, measured on the elevated plus maze (Finn et al., 2018). We found that all naïve mice in our 2018 study exhibited a PS-induced increase in anxiety-related behavior, consistent with results in male rats where both “Avoider” and “Non-Avoider” rats exhibited increased anxiety-related behavior after PS (Whitaker and Gilpin, 2015; Albrechet-Souza et al., 2020). Thus, PS-induced changes in anxiety-related behavior may not distinguish PS sensitivity subgroups (i.e., “sensitive” and “resilient” mice in our study; “Avoider” vs. “Non-Avoider” rats). Likewise, whereas male and female rats exhibited differences in active vs. passive coping behaviors during PS exposure, all rats showed enhanced behavioral reactivity to the PS-paired context (Ornelas et al., 2021). So, it is not known is whether associations between PS-induced change in anxiety-related behavior and other phenotypes differ in the PS sensitivity subgroups. It also is not known whether PS-enhanced drinking in “sensitive” male and female mice would be associated with a PS-induced increase in anxiety-related behavior or whether PS-enhanced drinking corresponded to a compensatory mechanism to offset anxiety-related behavior. Future studies will investigate this possibility.

Our retrospective determination of PS sensitivity subgroups revealed that BL ethanol intake was significantly lower in animals in the “sensitive” vs. “resilient” subgroup. This finding is similar to our earlier observation in females, where there were two separable BL subgroups, and PS-enhanced drinking was evident only in the subgroup of female mice with lower BLs (Finn et al., 2018). For the current studies, BL drinking was measured for up to 4 weeks and was very stable across time. So, it is unlikely that these subgroup differences in BL drinking reflect differences in stability of the measurement across time. It is notable that there was a divergent pattern of change in ethanol drinking after intermittent PS in the “sensitive” and “resilient” subgroups in both males and females, with a progressive increase in drinking after each PS in the “sensitive” subgroup and a consistent decrease in drinking after PS in the “resilient” subgroup (Figures 3A,B). Additionally, ethanol intake (g/kg) after PS4 was higher in the “sensitive” vs. “resilient” subgroup. Collectively, the different patterns of change in ethanol intake after PS as well as the higher g/kg intake after PS4 in the “sensitive” vs. “resilient” subgroup suggest that different mechanisms in response to stress may underlie the opposite drinking patterns. One possibility is that PS-enhanced drinking in the “sensitive” subgroup reflects an increase in drinking to offset PS-induced anxiety (as mentioned above). An alternate possibility, and one that may be congruent with the lower BL in the “sensitive” subgroup, is that PS history increases the rewarding properties and/or blunts the aversive properties of ethanol in the “sensitive” subgroup. Consistent with this idea, PS-enhanced ethanol self-administration in “Avoider” male rats was associated with resistance to quinine adulteration of the ethanol solution, considered a model of compulsive-like ethanol drinking (Edwards et al., 2013). A ten-fold higher concentration of quinine (0.025 vs. 0.0025%) was required to reduce ethanol self-administration in the “Avoider” vs. “Non-Avoider” rats and unstressed controls. A later study also documented that “Avoider” rats exhibited a reduction in the aversive properties of a moderate ethanol dose, when measured with a conditioned place aversion procedure (Schreiber et al., 2019). A study in C57BL/6J mice also determined that a history of PS produced resistance to quinine adulteration of an ethanol solution in male but not female mice (Shaw et al., 2020). Although PS-enhanced drinking was not measured, higher concentrations of quinine were required to reduce ethanol consumption vs. BL in PS-stressed males than in unstressed controls. Preliminary results from our lab suggest that there is resistance to quinine adulteration of a 10% ethanol solution in male mice from the “sensitive” vs. “resilient” subgroups (Helms and Finn, unpublished); the reduction in ethanol intake with 0.01% quinine was significantly greater in the “resilient” subgroup and unstressed controls than in the “sensitive” subgroup. Although females have not been tested yet, additional studies will determine whether “sensitivity” to PS-enhanced drinking is associated with a reduction in ethanol’s aversive properties.

Plasma CORT levels were measured as a bioassay for HPA axis responsivity after intermittent PS exposure and a 24 h abstinence after the final drinking session. There were several interesting findings. First, PS exposure significantly increased CORT levels vs. naïve, consistent with data from our and other laboratories, where various models of PS exposure significantly increased CORT levels (Mazor et al., 2009; Cozzoli et al., 2014; Whitaker and Gilpin, 2015; Finn et al., 2018; Shaw et al., 2020). Despite the fact that CORT levels in the naïve group were obtained upon euthanasia, the PS-induced increase in plasma CORT levels over naïve was 124 and 88% for animals in the “resistant” and “sensitive” subgroups, respectively. The highly significant and consistent PS-induced increase in CORT levels after various PS from our and other laboratories suggests that the different treatment of the naïve group did not confound interpretation of the present results. Second, the increase in CORT levels was similar across PS exposures. This finding combines with prior work to suggest that repeated PS exposures did not produce a sensitized CORT response, as levels were similar after the first and fourth PS exposure in the current and our prior work (Finn et al., 2018) and also were similar when repeated PS exposures were administered during adolescence and again during adulthood (Shaw et al., 2020). Third, the PS-induced increase in CORT levels was significantly greater in “resilient” vs. “sensitive” male and female mice. One interpretation of this result is that increased drinking after PS in “sensitive” mice may not be a direct consequence of the PS-induced increase in CORT levels. On the other hand, this result is consistent with the report that circulating ACTH and CORT concentrations were significantly higher after PS in “Non-Avoider” vs. “Avoider” male rats (Whitaker and Gilpin, 2015). It should be noted that an enhanced CORT response to PS was observed in “EBR” male rats (Cohen et al., 2007); however, animals in this “extreme behavioral response” model of PTSD have not been characterized for changes in ethanol drinking after PS exposure. Thus, the subpopulations of animals that exhibited PS-enhanced drinking (“Avoider” male rats and “sensitive” male and female mice in the present study) also exhibited a lower increase in ACTH and CORT levels after PS, suggestive of a dysregulation in the HPA axis response to stress in these animals. Fourth, the PS-induced increase in CORT levels was significantly higher in female vs. male mice, in harmony with other work (Mazor et al., 2009; Cozzoli et al., 2014; Finn et al., 2018; Shaw et al., 2020). In our experience, exposure to various stressors resulted in higher CORT levels in female vs. male mice, consistent with reports of sex differences in HPA axis responsivity to stress [reviewed in Bourke et al. (2012), Valentino et al. (2013a,b), Bangasser and Valentino (2014), Panagiotakopoulos and Neigh (2014), and references therein]. Finally, CORT levels remained elevated only in females following 24 h abstinence after the final drinking session; CORT levels in males were not different from values in naïve mice. Although we did not measure symptoms of withdrawal in the present studies, 24 h abstinence from ethanol consumption with several different drinking models has been shown to produce some somatic and negative affective symptoms in male and female rodents, with some reports of lower incidence of symptoms in female rodents [e.g., see review by Bloch et al. (2022) and references therein; Smith et al., 2016; Li et al., 2019]. Collectively, the CORT results suggest that there may be a greater dysregulation of the HPA axis following intermittent PS and a history of ethanol drinking in female vs. male mice.

Our study analyzed levels of proteins involved in regulation of stress responses at 24 h of abstinence following continuous ethanol drinking. We acknowledge that distinct changes in these protein levels could occur at different time points of the procedure due to plasticity associated with the effects of ethanol and PS (e.g., Brancato et al., 2021). Our prior work observed significant alterations in hippocampal protein levels of two proteins involved in synaptic plasticity (ARC or activity-regulated cytoskeletal protein and synaptophysin; Devaud et al., 2020). Nevertheless, Western blots confirmed sexually divergent and brain regional changes in levels of major proteins involved in the regulation of stress and anxiety-like responses. In female mPFC, levels of all four measured proteins were significantly higher in the “sensitive” vs. “resilient” subgroup. For CRF-R2, CRF-BP, and GR, these levels were also significantly higher than the naïve group. With the exception of CRF-BP, no increases in these stress-related proteins were observed in “sensitive” vs. “resilient” and naive groups in males. Despite no significant influence of sex on sensitivity to PS-enhanced drinking or to the PS-induced increase in CORT levels, only “sensitive” female mice responded with increased mPFC CRF-R2 and GR levels. It is likely that the higher CORT levels in females vs. males after 24 h abstinence, when protein levels were assessed, contributed to the significant upregulation of all 4 proteins in female mPFC as well as to the positive correlations between final CORT levels and each of the proteins examined. Changes in stress-related proteins in the hippocampus were less sex-dependent. In contrast to the mPFC, PS-enhanced drinking was not associated with changes in “sensitive” mice, but with sex-independent decreases in CRF-R1, CRF-R2, and GR in “resilient” mice. The difference in changes in the tested proteins in mPFC vs. hippocampus suggests that the “sensitivity” and “resilience” phenotypes involve these brain regions differentially. Thus, “sensitivity” is primarily associated with female-preferential increases in CRF-R1, CRF-R2, and GR in the mPFC. On the other hand, “resilience” is primarily associated with sex-independent decreases in CRF-R1, CRF-R2, and GR in the hippocampus. Considering the “sensitive” mice as a model of the vulnerable PTSD-AUD population, we suggest that special attention needs to be paid to increased markers of stress in these mice. Therefore, our findings suggest the need of potential targeting of CRF-R2 and GR in mPFC to counteract this sensitivity to PS-enhanced drinking. While no attempts of manipulating CRF-R2 receptors in mPFC to modulate ethanol intake have been performed yet, it is interesting to consider that CRF-R2 colocalize with dopamine D1R in mPFC and regulate glutamatergic activity in this region (Yarur et al., 2021). On the other hand, GR in mPFC has been shown to be affected in a rat model of alcohol dependence (Somkuwar et al., 2017), and a GR antagonist has been demonstrated to decrease alcohol seeking in alcohol-dependent rats and humans (Vendruscolo et al., 2015) and to decrease binge drinking in male and female mice (Savarese et al., 2020). Our results suggest the need for greater attention to potential sex differences in studies with GR antagonists.

Taking into consideration the potential treatments for individuals with PTSD-AUD comorbidity, it is notable that CRF-R1 was the least affected among stress-related proteins in the “sensitive” mice. Thus, the only increase in CRF-R1 in the “sensitive” mice in the present study was observed in the mPFC of female mice as compared to “resilient” females. This increase was not statistically significant when compared to naïve mice. On one hand, this observation suggests that the previously observed increase in CRF-R1 levels in PS-exposed alcohol-exposed mice in the hippocampus (Finn et al., 2018) could be related to the mild fluid restriction that was used in the previous study. Importantly, on the other hand, the lack of strong associations between sensitivity to PS-enhanced drinking and the CRF-R1 levels are in agreement with the limited efficacy of clinical approaches to target CRF-R1 in AUD patients (Spierling and Zorrilla, 2017). Therefore, our studies provide evidence that PS-enhanced drinking could be a translationally relevant model of PTSD-AUD comorbidity and call for potential targeting of other stress-related proteins in the treatment of harmful alcohol drinking patterns in this vulnerable population.

It needs to be specially noted that changes in CRF-BP appeared the most consistent between the subgroups. Specifically, CRF-BP showed increased levels in “sensitive” mice of both sexes in mPFC when compared to both “resilient” and naïve animals, and showed increased levels in the hippocampus of male “sensitive” mice vs. both “resilient” and naïve, while showing no changes in the hippocampus of female “sensitive” mice vs. the other groups. It is possible that the higher CRF-BP levels in the “sensitive” subgroup contribute to the significantly lower CORT concentrations after PS in the “sensitive” vs. “resilient” subgroup, as one role of CRF-BP is to bind and sequester circulating CRF and the urocortins (e.g., Westphal and Seasholtz, 2006). Both stress and glucocorticoids can increase CRF-BP expression [reviewed in Westphal and Seasholtz (2006)], so the significant increase in CRF-BP levels in the “sensitive” subgroup vs. naïve in mPFC and male hippocampus may reflect the combination of intermittent PS exposure with enhanced ethanol drinking. On the other hand, CRF-BP may not only function to sequester peptides, but has also been proposed to enhance neuronal activity by interactions with CRF-R2 (Ungless et al., 2003; Haass-Koffler, 2018), or even to facilitate the presence of CRF-R2 receptors on the cell surface (Slater et al., 2016). Therefore, it is yet difficult to interpret the functional significance of changes in CRF-BP. For example, knockout of CRF-BP has been reported to both increase and decrease ethanol consumption (Haass-Koffler et al., 2016; Ketchesin et al., 2016). On the other hand, CRF-BP associating with CRF-R2 in the ventral tegmental area can act to modulate binge drinking (Albrechet-Souza et al., 2015). CRF-BP undergoes proteolytic cleavage into two smaller proteins that likely contribute to its opposing roles; CRF-BP (27kD) binds CRF and is responsible for neutralizing its effects, and CRF-BP (10kD) has an excitatory role by potentiating CRF-R2 signaling (Haass-Koffler et al., 2016; Haass-Koffler, 2018). Thus, these dual roles of CRF-BP would contribute differently to stress adaptation and likely produce mixed effects in models of stress and ethanol drinking. In either case, studies are needed to evaluate the potential role of PS-induced changes in CRF-BP in HPA regulation and enhanced ethanol intake.

In conclusion, we developed an animal model where repeated exposure to predator odor as a traumatic stress significantly increased later ethanol drinking in a proportion of PS-sensitive male and female mice. The heterogeneity of responses is consistent with results from studies utilizing other predator odors as models of PTSD in rodents. Our initial characterization of “sensitivity” and “resilience” to PS-enhanced drinking revealed that “sensitivity” was associated with a blunted CORT response to PS in both males and females, but also with sex-dependent differences in proteins related to stress-regulating systems in mPFC and hippocampus. Despite large differences in sample size between males and females in the full dataset (males > females), similar results in drinking measures and CORT responses were obtained with the dataset of animals utilized for the Western blot study when group size was similar in males and females. Future studies will use this model to examine sex differences in biological and molecular mechanisms that confer susceptibility for increased drinking following traumatic stress so that the information can guide the development of new interventions for the treatment of PTSD-induced AUD, which likely differ in males and females.

## Data Availability Statement

The raw data supporting the conclusions of this article will be made available by the authors, without undue reservation.

## Ethics Statement

The animal study was reviewed and approved by Institutional Animal Care and Use Committee at VA Portland Health Care System.

## Author Contributions

DF, AR, and LD contributed to conception and study design. DF, MH, and MN conducted the drinking studies. MH and MN dissected prefrontal cortex and hippocampal tissue. MA and LD isolated protein and prepared samples for Western Blot analysis. MA conducted the Western Blots and MA and LD quantified the protein levels. MH conducted preliminary analysis of drinking data and DF, MH, and MN identified animals for the Western Blot analysis. DF, MN, MH, and AR participated in final analysis of drinking data. MH, DF, and AR contributed to analysis of the Western Blot data. LD and AR assisted in the interpretation of the Western Blot analyses. DF and AR wrote the first drafts of the manuscript, but MA wrote sections of the Methods and Results. All authors contributed, read, and approved the final version of manuscript.

## Conflict of Interest

The authors declare that the research was conducted in the absence of any commercial or financial relationships that could be construed as a potential conflict of interest.

## Publisher’s Note

All claims expressed in this article are solely those of the authors and do not necessarily represent those of their affiliated organizations, or those of the publisher, the editors and the reviewers. Any product that may be evaluated in this article, or claim that may be made by its manufacturer, is not guaranteed or endorsed by the publisher.
