# Supplementary material for: Sensitivity and Resilience to Predator Stress-Enhanced Ethanol Drinking Is Associated With Sex-Dependent Differences in Stress-Regulating Systems
Source: Front Behav Neurosci. 2022 May 11;16:834880. doi: 10.3389/fnbeh.2022.834880 (PMC9132579; doi:10.3389/fnbeh.2022.834880)

Sensitivity and Resilience to Predator Stress-Enhanced Ethanol Drinking is Associated with Sex-Dependent Differences in Stress-Regulating Systems

Mehrdad Alavi, Andrey E. Ryabinin, Melinda L. Helms, Michelle L. Nipper, Leslie L. Devaud, and Deborah A. Finn

Supplemental Material

Supplemental Figure Legends:

Supplemental Figure 1. Representative images of immunoblots analyzing corticotropin releasing factor receptor 1 (CRF-R1) for each sex and brain region. The top panels reflect images for medial prefrontal cortex (mPFC), whereas the bottom panels reflect images for hippocampus (Hipp). The protein standard ladder (middle) separates the images for females (left) and males (right). Numbers correspond to molecular weights of standards in kilodaltons (kDa). Note the single band on the blot incubated with the antibody for CRF-R1 that corresponded in size to 52 kDa (annotated with arrow). The red signal corresponded to over-saturation of signal, which was not quantified.

Supplemental Figure 2. Representative images of immunoblots analyzing corticotropin releasing factor receptor 2 (CRF-R2) for each sex and brain region. The top panels reflect images for medial prefrontal cortex (mPFC), whereas the bottom panels reflect images for hippocampus (Hipp). The protein standard ladder (middle) separates the images for females (left) and males (right). Numbers correspond to molecular weights of standards in kilodaltons (kDa). Note that blots incubated with the antibody for CRF-R2 showed several bands reflecting alternate isoforms of the protein; test blots and validation studies made it possible to choose the correct band with a good level of confidence. The correct band corresponded in size to 44 kDa (annotated with arrow).

Supplemental Figure 3. Representative images of immunoblots analyzing corticotropin releasing factor binding protein (CRF-BP) for each sex and brain region. The top panels reflect images for medial prefrontal cortex (mPFC), whereas the bottom panels reflect images for hippocampus (Hipp). The protein standard ladder (middle) separates the images for females (left) and males (right). Numbers correspond to molecular weights of standards in kilodaltons (kDa). Note that blots incubated with the antibody for CRF-BP showed several bands reflecting alternate isoforms of the protein; test blots and validation studies made it possible to choose the correct band with a good level of confidence. The correct band corresponded in size to 36 kDa (annotated with arrow). The strong saturated band (red signal) reflects carry over from prior blotting with the antibody against the glucocorticoid receptor (GR, ~97 kDa). The large size difference in proteins for CRF-BP and GR made it possible to blot first with the antibody for GR, strip, and then blot with the antibody for CRF-BP. Because GR gave a much stronger signal than CRF-BP, this accounted for the appearance of the saturated red band close to 100 kDa on the CRF-BP immunoblots, even after stripping to remove the GR antibody.

Supplemental Figure 4. Representative images of immunoblots analyzing glucocorticoid receptor (GR) for each sex and brain region. The top panels reflect images for medial prefrontal cortex (mPFC), whereas the bottom panels reflect images for hippocampus (Hipp). The protein standard ladder (middle) separates the images for females (left) and males (right). Numbers correspond to molecular weights of standards in kilodaltons (kDa). Note the strong band on the blot incubated with the antibody for GR that corresponded in size to 97 kDa (annotated with arrow).

Supplemental Figure 1

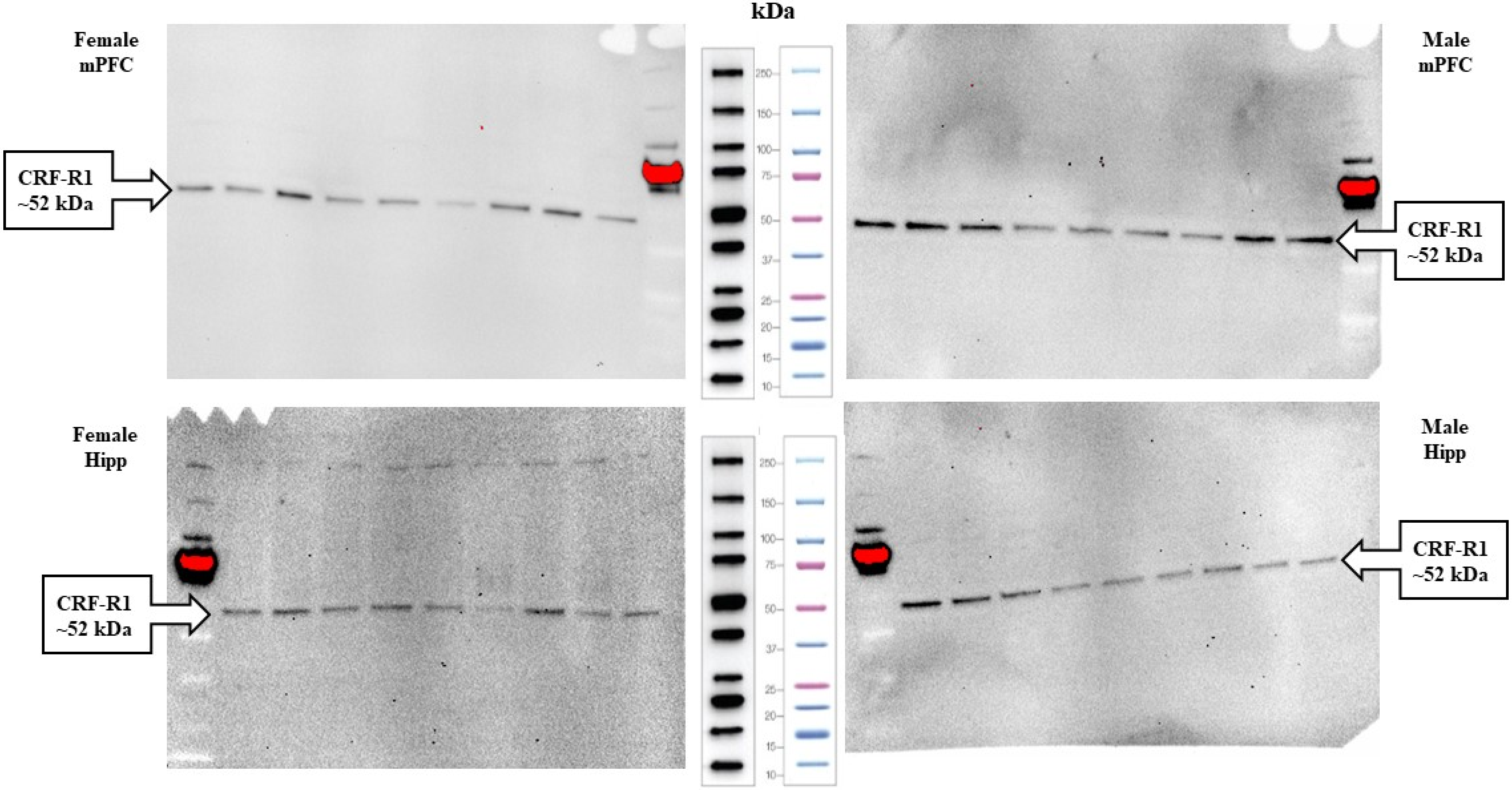

Supplemental Figure 2

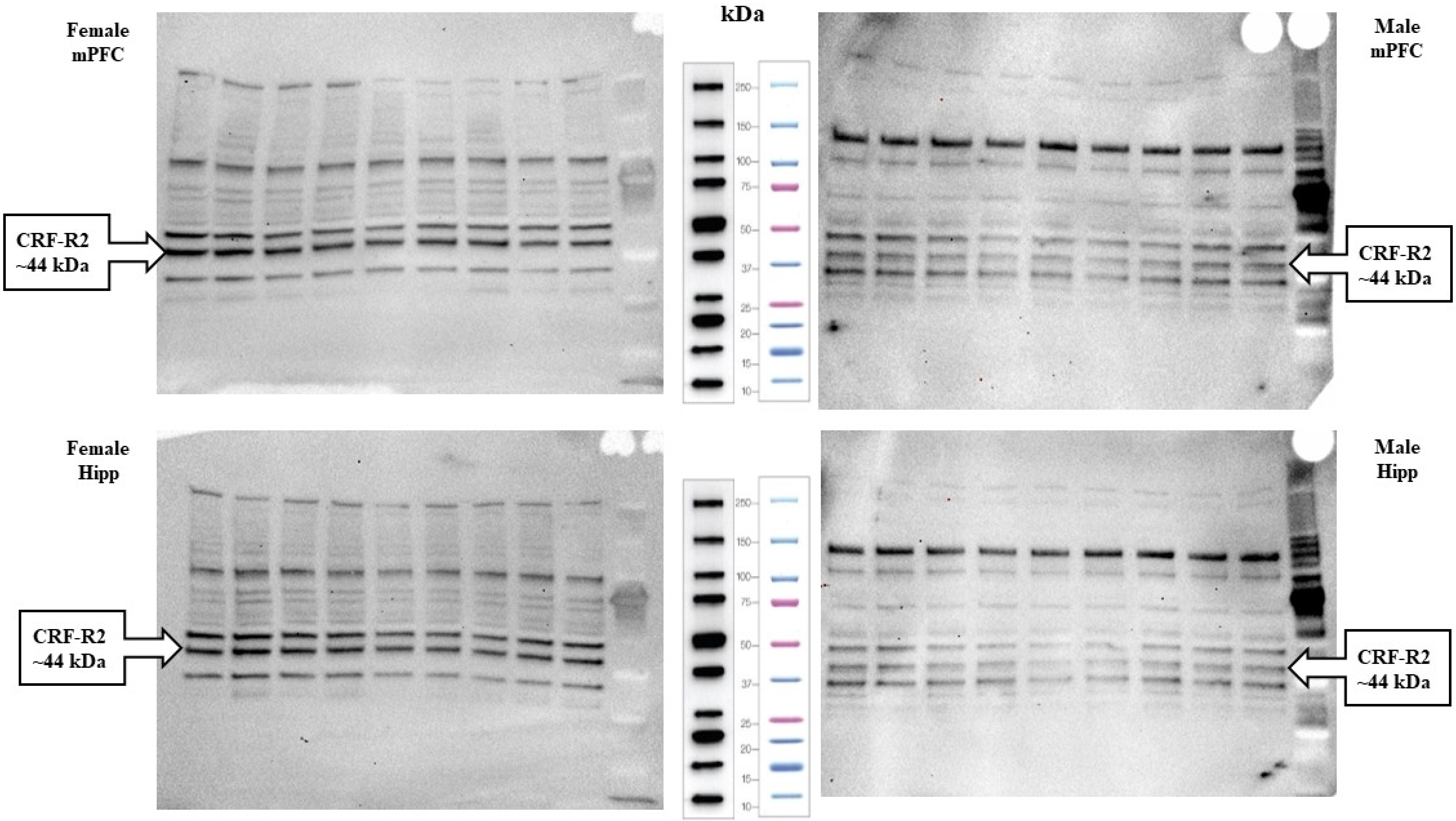

Supplemental Figure 3

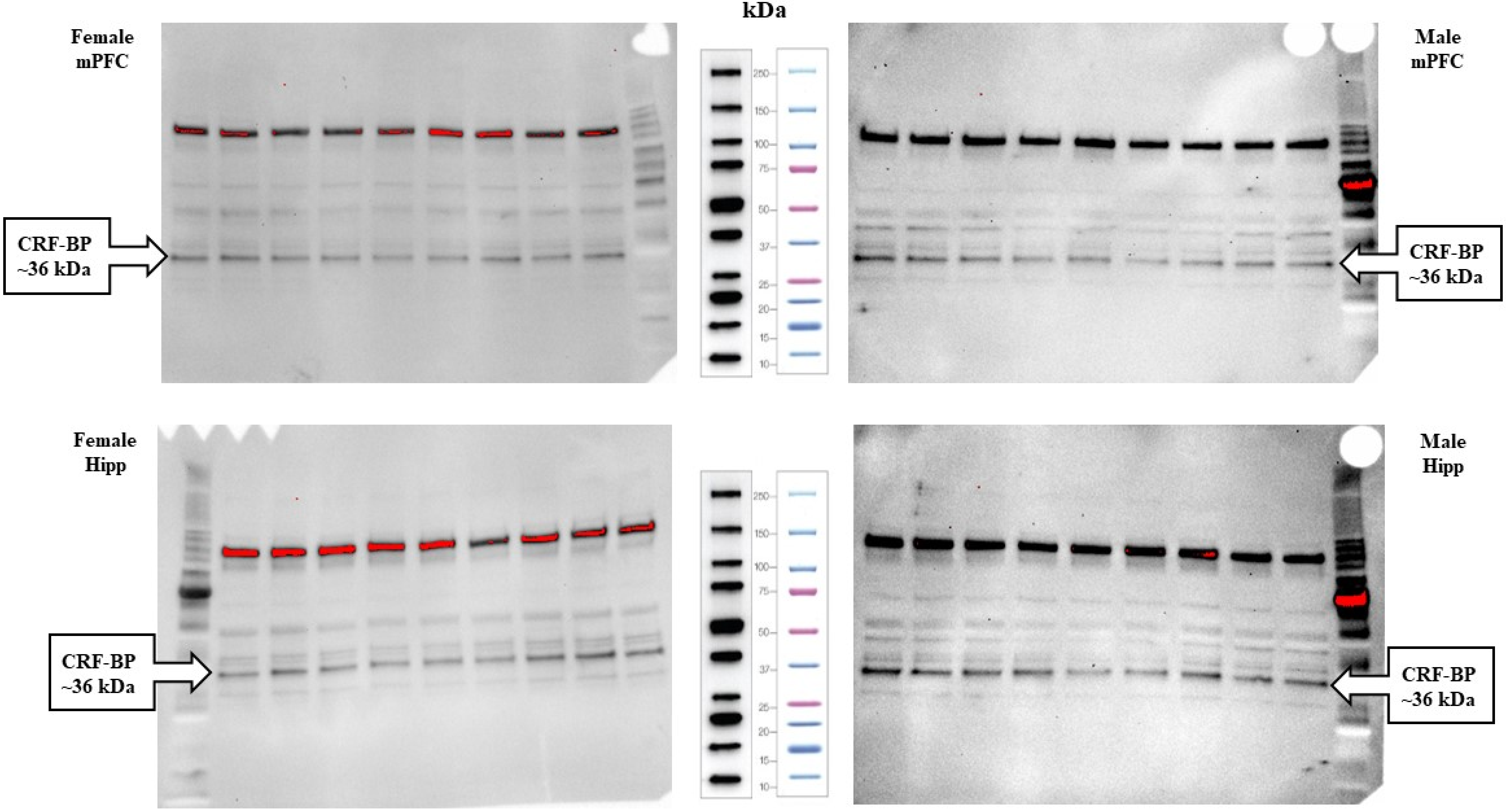

Supplemental Figure 4

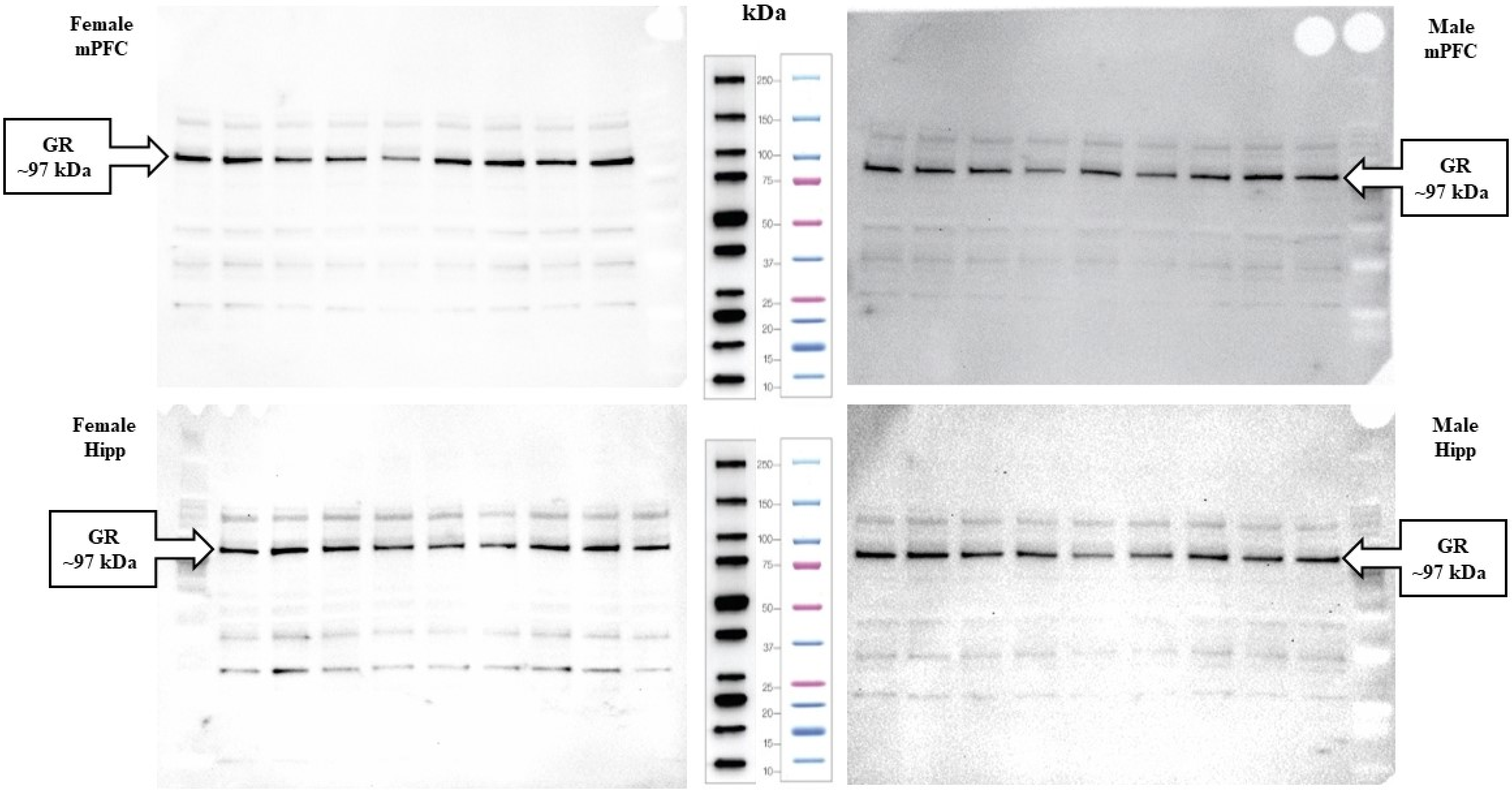

Supplement: Supplementary file 1 [file Data_Sheet_1.pdf]
